# SMILES-based degree molecular descriptors and machine learning for QSPR modeling of anti-alkaptonuria drugs

**DOI:** 10.3389/fchem.2026.1845927

**Published:** 2026-06-15

**Authors:** Guorong Wu, Muhammad Waheed Rasheed, Samirah Alsulami

**Affiliations:** 1 School of Mathematics and Statistics, Chongqing Sanxia University of Science and Technology, Chongqing, China; 2 Faculty of Computer Science and Information Technology, Superior University Lahore, Lahore, Pakistan; 3 Department of Mathematics and Statistics, Faculty of Science, University of Jeddah, Jeddah, Saudi Arabia

**Keywords:** alkaptonuria, gradient boosting regression, QSPR analysis, random forest regression, smiles

## Abstract

Quantitative Structure-Property Relationship (QSPR) modelling provides an efficient computational framework for predicting physicochemical properties of drug molecules when experimental data are limited. In this study, we investigate the predictive capability of degree-based topological indices (TIs) derived from SMILES (Simplified Molecular Input Line Entry System) representations for modelling physicochemical properties of anti-alkaptonuria drugs. Nine representative compounds, including Nitisinone, Ascorbic Acid, Ibuprofen, Naproxen, Paracetamol, Tramadol, Methotrexate, Sulfasalazine, and Glucosamine, were analysed using several molecular descriptors such as molecular weight, logP, hydrogen bond donors and acceptors, rotatable bonds, and polar surface area. A total of 58 regression models were developed using Linear Regression (LR) and two machine learning algorithms, Random Forest (RF) and Extreme Gradient Boosting (XGBoost, abbreviated XGB). Model performance was evaluated using Mean Absolute Error (MAE), Mean Squared Error (MSE), Root Mean Squared Error (RMSE), and the coefficient of determination $R^{2}$ . The results demonstrate that machine learning models significantly outperform classical regression, with XGB achieving the most accurate and stable predictions for the investigated physicochemical properties. This study introduces a machine learning-driven QSPR framework that integrates SMILES-derived degree-based topological indices with ensemble learning techniques for predicting physicochemical properties of anti-alkaptonuria drugs. The proposed approach demonstrates improved predictive performance on small datasets and highlights the effectiveness of combining graph-theoretic molecular descriptors with advanced machine learning methods.

## 1 Introduction

Alkaptonuria is a rare autosomal recessive genetic disorder caused by deficiency of the enzyme homogentisic 1,2-dioxygenase (HGD). The HGD gene (located on chromosome 3q13.33) is inherited in an autosomal recessive pattern, meaning two mutated copies of the gene are required for the disease to manifest. The liver, kidneys, prostate, small intestine, and colon are all places where the HGD gene is highly expressed (Ranganath et al., 2013). The conversion of homogentisic acid (HGA) into malate and acetoacetate is a necessary step in the metabolic process of tyrosine, which is facilitated by this enzyme. The liver produces an excessive amount of homogentisic acid, which, in the absence of HGD, undergoes oxidation and transforms into ochronotic pigment polymer. The development of systemic illness is caused by the accumulation of this pigment in various tissues. An ochronosis is the name given to this procedure.

When it came to human genetic illnesses, alkaptonuria was one of the first to be discovered to adhere to the rules of mendelian recessive inheritance (Garrod, 1996). In the realm of history, it was utilised by Archibald Garrod in his croonian lectures in the year 1908 to highlight the fundamental concepts that underlie “inborn errors of metabolism”. On the other hand, the Egyptian mummy known as Harwa is said to have been the first clinical example of alkaptonuria, and it dates back to the year 1500 BC (Stenn et al., 1977). With its roots in the arabic word “alkali,” “alkaptonuria” was first encountered (O’brien et al., 1963). In addition, Boedeker initially came up with the term in the year 1859 after seeing peculiar diminishing qualities in the urine of patients (Boedeker, 1859). Under the microscope, Virchow found ochronosis in 1866 when HGA pigment looked pale brownish yellow (ochre-like) (Virchow, 1866).

Although it is uncommon, alkaptonuria affects people all over the globe. The estimated incidence of alkaptonuria is approximately 1 in 100,000 to 1 in 250,000 live births worldwide. One instance per million people is the disease’s prevalence in the US. U.S. data from the AKU Society and the develop AKUre Consortium indicate that 92 people suffer with alkaptonuria. Slovak and Dominican news outlets mostly cover it (Ranganath et al., 2011). Although it is somewhat more common among the african population, it is present in all racial groups. The disease affects both sexes equally, but it is more severe in men.

The para-hydroxylated ring shape of tyrosine is the culprit in its catabolic process. The production of melanin, hormones, and a wide variety of proteins all rely on tyrosine, an amino acid that plays an important role in many bodily processes. Nevertheless, the majority of the tyrosine that humans take in does not go to waste; it is transformed into acetoacetate and malate.

**FIGURE 1:**
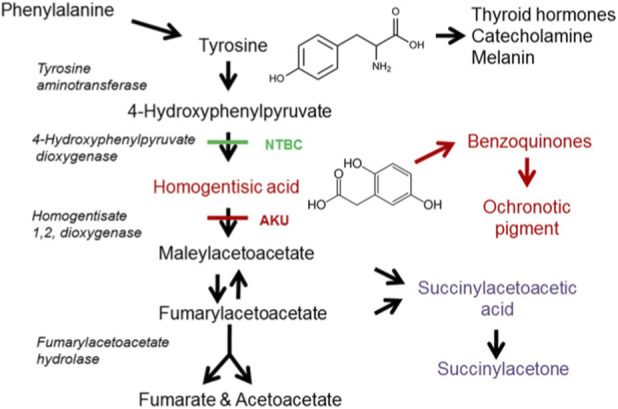
The tyrosine degradation pathway.

The metabolism of tyrosine is facilitated by the enzyme homogentisic 1,2-dioxygenase (HGD) (Phornphutkul et al., 2002). Its significance in the tyrosine metabolic pathway is seen in Figure 1. A crucial step in the breakdown of tyrosine, its principal purpose is to convert homogentisic acid (HGA) into maleylacetoacetate. Located on chromosome 3q13.33 in the human genome, HGD is a protein of 445 amino acids.

But the HGD enzyme stops working as it should when a mutation happens in the HGD gene. One of the symptoms of alkaptonuria is an abnormal buildup of homogentisic acid (HGA) in the body; this condition is caused by a genetic mutation that makes the HGD enzyme ineffective.

The metabolic disorder known as alkaptonuria is marked by an abnormal accumulation of homogentisic acid (HGA) in the bloodstream. This leads to symptoms such as black urine, tendinitis, kidney and prostate stones, joint roughness from HGA deposition, ankylosis, kyphosis, and stiffened heart valves. The presence of oxygen, perspiration, and earwax, in addition to the accumulation of HGA in connective tissues such as cartilage, might amplify symptoms (Pa, 1951).

The condition known as alkaptonuria can be addressed by the use of both targeted therapy and symptomatic treatment. The metabolic route that is responsible for the formation of HGA is significantly inhibited by the most effective disease-modifying medication, nitisinone (Teke Kisa et al., 2022). The antioxidant capacity of ascorbic acid, often known as vitamin C, may help slow down the damage that HGA causes to tissues (Wolff et al., 1989). In most cases, nonsteroidal anti-inflammatory drugs (NSAIDs) like ibuprofen and naproxen are used to alleviate the symptoms of pain and inflammation. Paracetamol, on the other hand, is a more gentle option. The usage of tramadol could be necessary in cases of severe pain (Alhelal et al., 2023). The nine compounds analyzed in this study include drugs used in alkaptonuria management, though their primary indications vary. Nitisinone is the only disease-modifying therapy that reduces HGA production. Ascorbic acid (vitamin C) is used as an antioxidant supplement to slow tissue damage. Ibuprofen, naproxen, paracetamol (acetaminophen), and tramadol are analgesics/anti-inflammatory drugs used for symptom management (pain and inflammation). Methotrexate and sulfasalazine are immunomodulators sometimes used for associated arthritis, though evidence for alkaptonuria-specific arthropathy is limited. Glucosamine is a dietary supplement with unproven efficacy for joint function in this condition. Thus, these compounds represent a mixture of disease-modifying, symptomatic, and supportive agents rather than a homogeneous set of ‘anti-alkaptonuria’ drugs.

The process of designing and discovering new drugs is notoriously difficult, time-consuming, and costly. Consequently, academics have focused on finding ways to optimise this process, particularly when resources are limited or there are emergencies. The multidisciplinary subject of chemical graph theory is used for the study of molecular structures and the discovery of relationships between different events, activities, and qualities. Here, the structural formula of a chemical compound is depicted by a molecular graph, where the atoms are the nodes and the chemical bonds are the edges. Topological indexes are one of the new tools provided by chemical graph theory for analysing chemical structures. These indices are real numbers that describe the structure and special features of molecular graphs (Kulli, 2020). Topological indices have been used in a plethora of research to examine drug structures and molecular graphs (Ghods and Tousi, 2021; Tousi and Ghods, 2024). Quantitative Structure-Property Relationship (QSPR) models are a cornerstone for investigating the connection between the topological indices of a material and its physicochemical characteristics. These topological indicators and physical and chemical qualities are investigated in models by regression analysis. Quantitative Structure-Activity Relationships (QSAR) research has extensively used topological indices to analyse drug structures (Havare, 2022).

Recent advances in graph-theoretic chemistry have further demonstrated the effectiveness of topological descriptors in predicting physicochemical and thermodynamic properties of molecular systems. Foundational contributions to the development and chemical interpretation of degree-based topological indices were established by Gutman (Ismail et al., 2025). Degree- and irregularity-based descriptors have also been successfully applied to benzenoid systems for analyzing structural and physicochemical behavior (Chu et al., 2021). Furthermore, regression-based QSPR investigations involving Seidel energies and thermodynamic properties have shown the predictive capability of graph-energy descriptors in chemical modeling (Bilal et al., 2025). Extended graph energies have similarly been utilized for thermodynamic property prediction through QSPR frameworks (Gutman, 2013). More recently, energy-based molecular descriptors have demonstrated strong applicability in QSPR modeling and physicochemical property estimation (Naz et al., 2025). These studies collectively highlight the growing importance of graph-theoretic descriptors and machine learning approaches in modern computational chemistry and provide additional theoretical motivation for the present study.

Out of the limitations of standard graph theory, the present study proposes an auxiliary approach to assess the interaction between substructural pieces in molecular structures. Subgraphs in a complicated chemical network may be fully identified using this method, which makes use of graph theory and SMILES notation (Weininger, 1988). With the use of SMILES, which is based on atom-specific connection patterns produced from input structural data, “atom-specific” discrimination is theoretically feasible, including hybrids and positional context within a molecule. This capability makes graph theory both fast and effective (Weininger et al., 1989). In doing so, it constructs a graph containing molecular connection data, associating chemical information with each subgraph that corresponds to a chemically significant substructural component in SMILES.

Although traditional linear regression is appropriate for small datasets, tree-based ensemble methods such as Random Forest and XGB were explored for two reasons. First, the relationship between topological indices and physicochemical properties is not guaranteed to be linear; ensemble methods can capture potential nonlinear interactions. Second, this study serves as a proof-of-concept to evaluate whether modern ML methods can extract meaningful patterns from extremely small chemical datasets when experimental data are scarce. By training many decision trees and then combining their results to reduce overfitting and optimize generalization, Random Forest (RF), an ensemble learning method, improves the reliability of predictions. XGB (XGB), a robust gradient boosting method, successively improves weak learners to enhance predictions, making it suitable for capturing complex chemical interactions. The most effective method for capturing the essential pharmacological features was determined by comparing the models’ predictions. This study contributes to medication design and treatment optimization in alkaptonuria therapies by helping to select the best suitable prediction model (Bilal et al., 2025; Gutman, 2013). However, the reader should note that results on n = 9 require cautious interpretation, and cross-validation is essential to avoid overstatement of performance (see Section 2.5).

Recent studies have applied topological indices and machine learning techniques to QSPR modeling across various drug classes. Havare (2022) investigated degree-based indices for COVID-19-related compounds using linear regression, while Ahmed et al. (2024) explored supervised learning approaches for anti-HIV drugs. However, to the best of our knowledge, no study has specifically applied ensemble learning methods (Random Forest and XGBoost) to SMILES-derived degree-based topological indices for alkaptonuria-related compounds. The contributions of this study are threefold: (1) it presents the first QSPR analysis of anti-alkaptonuria compounds using degree-based topological indices; (2) it provides a systematic comparison of linear regression with ensemble methods (Random Forest and XGBoost) on the same dataset; and (3) it demonstrates the applicability of machine learning-based prediction in ultra-rare disease contexts, where experimental data are highly limited. Importantly, the leave-one-out cross-validation (LOOCV) results reported in this study provide more realistic estimates of predictive performance, addressing the tendency toward overly optimistic results in small-sample QSPR modeling.

## 2 Preliminaries

**FIGURE 2:**
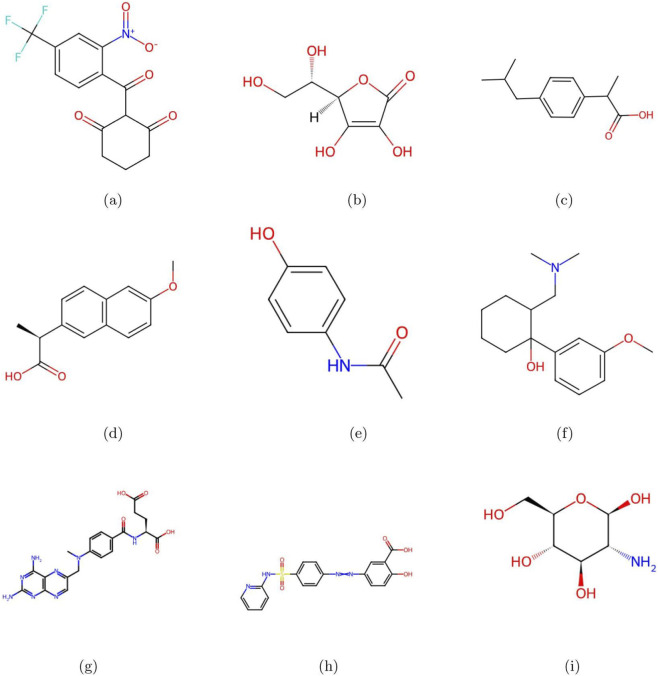
Molecular graph of alkaptonuria medications. **(a)** Nitisinone. **(b)** Ascorbic Acid. **(c)** Ibuprofen. **(d)** Naproxen. **(e)** Paracetamol. **(f)** Tramadol. **(g)** Methotrexate. **(h)** Sulfasalazine. **(i)** Glucosamine.

A graph G = (V, E) is a pair that consists of a set of edges E and a set of vertices V that are not empty to contain any elements. Throughout this article, every single graph that is discussed is straightforward, connected, and undirected. For this purpose, we make use of the chemical structure shown in Figure 2. Vertices are used to represent the atoms in these graphs, while edges are used to symbolise the bonds that connect them to one another. Degree-based topological indices capture information about molecular size, branching, and connectivity patterns. However, they do not encode three-dimensional geometry, electronic distribution, or stereochemistry. For QSPR modeling of simple physicochemical properties such as molecular weight and LogP, degree-based indices provide sufficient structural information. For more complex properties (e.g., binding affinity, metabolic stability), additional descriptors (3D, electronic, or pharmacophoric) would be necessary. This study is limited to degree-based indices as a proof-of-concept.

### 2.1 Degree-based descriptors

A numerical description of the structure of a molecular network, derived from the degrees (the number of connections) of its atoms (vertices), is called a degree-based topological index. A degree-based topological index (TI) is computed as:

$$TI\left(G\right)=\underset{uv\in E}{\sum }f\left(d_{u},d_{v}\right),$$

where $f\left(d_{u},d_{v}\right)$ is a symmetric function defining the relationship between degrees of adjacent vertices.

Some of the degree-based topological indices are defined as follows:

- Randic index

Randic index was defined by Randic (Randic, 1975):

$$R\left(G\right)=\underset{uv\in E\left(G\right)}{\sum }\frac{1}{\sqrt{d\left(u\right)\cdot d\left(v\right)}}.$$

ii. Harmonic index

Harmonic index was defined by Fajtlowicz (Fajtlowicz, 1988):

$$H\left(G\right)=\underset{uv\in E\left(G\right)}{\sum }\frac{2}{d\left(u\right)+d\left(v\right)}.$$

iii. Sum connectivity index

Sum connectivity index was defined by Zhou and Trinajstic (Zhou and Trinajstic, 2010):

$$\text{SCI}\left(G\right)=\underset{uv\in E\left(G\right)}{\sum }\frac{1}{\sqrt{d\left(u\right)+d\left(v\right)}}.$$

iv. First and second KCD indices

First and second KCD indices were defined by Mirajkar and Morajkar (Zhou and Trinajstic, 2010):

$${\text{KCD}}_{1}\left(G\right)=\underset{e=uv\in E\left(G\right)}{\sum }\left(d\left(u\right)+d\left(v\right)+d\left(e\right)\right),$$

$${\text{KCD}}_{2}\left(G\right)=\underset{e=uv\in E\left(G\right)}{\sum }\left(d\left(u\right)+d\left(v\right)\right)\cdot d\left(e\right).$$

Here, d(u) and d(v) denote the degrees of the vertices u and v , respectively, and d(e) denotes the degree of the edge e=uv .

### 2.2 QSPR model

A Quantitative Structure-Property Relationship (QSPR) model is mathematically defined as follows (Hui et al., 2023):

$$y=f\left(x_{1},x_{2},\ldots ,x_{n}\right),$$

where f is a function representing the relationship between the independent variables x (e.g., topological indices) and the dependent variable y (a physicochemical property).

In the case of a linear relationship, the model simplifies to:

y=a+bx,

where a is the regression constant and b is the regression coefficient. For nonlinear relationships, more sophisticated regression techniques such as machine learning algorithms are applied to capture complex patterns in the data.

The subsequent section provides key details about the machine learning methods employed in this study.

### 2.3 Machine learning (ML)

Machine learning refers to the field that studies how to program computers to interpret data automatically, without any human intervention. The greater the amount of data, the more capable the computers get. Organizations are increasingly embracing ML as fresh datasets continue to flood the market, demonstrating time and time again that these approaches can provide remarkable results. Studying how computers learn and behave has shown that they can pick up additional skills on their own. Mathematicians and computer scientists with access to such massive datasets have developed several approaches to answering data science concerns (Mahesh, 2020). The search for a universal ML algorithm continues. A problem’s optimal method or model, as well as the number of variables used to solve it, are both affected by its unique specifications. Algorithms like Naive Bayes, Decision trees, and Support Vector Machines are commonly used in ML.

#### 2.3.1 Supervised machine learning

Supervised learning is a subfield of machine learning within AI that allows computers to learn representational training data to create input-output mappings automatically (Ahmed et al., 2024). Algorithms such as Extreme Gradient Boosting (XGB) and Random Forest Algorithm (RF) are useful in predicting complicated nonlinear interactions using molecular data in drug development. Ensemble methods, such as RF building multiple decision trees and XGB sequentially optimizing tree-based models, achieve better predictive power toward complex biological interactions. Their success is because they effectively capture the high-dimensional patterns, feature dependencies, and nonlinear correlations that characterize pharmaceutical research. Random Forest, with its parallel tree construction, achieves relatively lower performance, whereas gradient-boosted trees from XGB typically offer state-of-the-art performance at higher computational complexities.

### 2.4 Error metrics

In regression analysis, three key metrics are commonly used to evaluate model performance: Mean Absolute Error (MAE), Mean Squared Error (MSE), and Root Mean Squared Error (RMSE).

#### 2.4.1 Mean absolute error (MAE)

MAE calculates the average absolute difference between predicted and actual values:

$$\text{MAE}=\frac{1}{N}\overset{N}{\underset{i=1}{\sum }}\left|y_{i}-{\hat{y}}_{i}\right|.$$

This metric provides a robust and easily interpretable measure by treating all errors equally, making it less sensitive to outliers.

#### 2.4.2 Mean squared error (MSE)

MSE computes the average of the squared differences between actual and predicted values:

$$\text{MSE}=\frac{1}{N}\overset{N}{\underset{i=1}{\sum }}{\left(y_{i}-{\hat{y}}_{i}\right)}^{2}.$$

The squaring process emphasizes larger errors, making MSE more sensitive to outliers. However, it is particularly useful during model training because of its differentiability.

#### 2.4.3 Root mean squared error (RMSE)

RMSE is defined as the square root of MSE:

$$\text{RMSE}=\sqrt{\frac{1}{N}\overset{N}{\underset{i=1}{\sum }}{\left(y_{i}-{\hat{y}}_{i}\right)}^{2}}.$$

This metric maintains the quadratic weighting of errors but expresses them in the original units of the target variable, offering a balance between sensitivity to large errors and ease of interpretation (Yang et al., 2005; Zaid, 2015).

#### 2.4.4 Interpretation

The choice among these metrics depends on the specific modeling objectives:

- MAE is often preferred when resistance to outliers is important.
- MSE is commonly used during training due to its mathematical properties.
- RMSE is frequently selected for final performance reporting as it provides an intuitive sense of average error in the same units as the predicted variable.

In the equations above:

-
$y_{i}$ represents the actual value (ground truth),
-
${\hat{y}}_{i}$ denotes the predicted value (model output),
-
N is the total number of data points in the dataset,
-
$R^{2}$ is the coefficient of determination.

### 2.5 Model validation strategy

Due to the limited dataset size (n = 9 compounds), a separate held-out test set was not feasible. Therefore, model performance was evaluated using leave-one-out cross-validation (LOOCV). In LOOCV, each model is trained on eight compounds and tested on the remaining one compound, repeated 9 times so that every compound serves as the test set exactly once. The reported performance metrics (MAE, MSE, RMSE, ${\text{R}}^{2}$ ) represent the average across all nine validation folds. This approach provides a more realistic estimate of generalizability than reporting training-set performance.

Importantly, all preprocessing and model evaluation steps were conducted within the leave-one-out cross-validation (LOOCV) framework to avoid data leakage. For each iteration, the model was trained exclusively on the training subset and subsequently evaluated on the held-out compound. No information from the test sample was incorporated during model fitting or prediction. The LOOCV procedure was implemented using the LeaveOneOut splitter together with cross_val_predict and related validation utilities from scikit-learn (version 1.0.2), ensuring an unbiased assessment of predictive performance.

## 3 Discussion of results

This section presents the main conclusions drawn from our investigation. The physical properties of alkaptonuria drugs are taken from a dataset that is accessible to the public. Information about the PubChem database may be found in Table 1 of the Appendix section. Predictive modeling makes use of the Random Forest algorithm (RF) and extreme gradient boosting (XGB), whereas linear regression (LR) models rely on backward-elimination feature selection to forecast all physical properties. Key predictive elements, such as sum degree-based topological indices (TIs) that control the model’s performance, may be identified with the use of both RF and XGB. To forecast the desired physical characteristics of alkaptonuria medications, these TIs have been entered into RF and XGB methods. Using gradient boosting and supervised machine learning, these models successfully extract intricate relationships and patterns from the data.

**TABLE 1:** Physicochemical properties of alkaptonuria-related compounds used in this study.

Drug name	MW (g/mol)	LogP^†^	H-bond donors	H-bond acceptors	Rotatable bonds	PSA (Å^2^)
Nitisinone	329.23	2.5	0	5	3	72.7
Ascorbic acid	176.12	−1.9	4	6	2	107.2
Ibuprofen	206.28	3.7	1	2	4	37.3
Naproxen	230.26	3.2	1	3	4	46.5
Paracetamol	151.16	0.5	2	3	2	49.3
Tramadol	263.38	2.9	1	3	4	32.7
Methotrexate	454.44	−1.8	6	12	8	175.9
Sulfasalazine	398.39	2.1	3	8	6	121.3
Glucosamine	179.17	−3.0	5	6	5	119.3

An important numerical number that predicts a given physical attribute is the sum degree-based topological index (TI), which has been retrieved from the converted chemical graph structure of each medication. The chemical graph that was transformed is shown in Figure 2. In this work, the structural data collected from the SMILES of the nine alkaptonuria medicines were used to determine their sum degree-based TIs. Table 2 no longer displays the structural information. The TIs provide insight into the impact of molecular connection patterns on specific physical properties. Table 3 displays the total number of degree-based TIs.

**TABLE 2:** SMILES strings and molecular formulas.

Drug name	SMILES	Molecular formula
Nitisinone	O=C1C(C(C2 = CC = C(C(F)(F)F)C=C2 [N+]([O-]) = O) = O)C(CCC1) = O	${\text{C}}_{14}{\text{H}}_{10}{\text{F}}_{3}{\text{NO}}_{5}$
Ascorbic acid (vit C)	C(C(=O)C (=O)O)O	${\text{C}}_{6}{\text{H}}_{8}{\text{O}}_{6}$
Ibuprofen	CC(C)CC1 = CC = C(C=C1)C(C)C (=O)O	${\text{C}}_{13}{\text{H}}_{18}{\text{O}}_{2}$
Naproxen	CC(=O)C1 = CC = C(C=C1)C(C)CC(=O)O	${\text{C}}_{14}{\text{H}}_{14}{\text{O}}_{3}$
Paracetamol	CC(=O)NC1 = CC = C(C=C1)O	${\text{C}}_{8}{\text{H}}_{9}{\text{NO}}_{2}$
Tramadol	CN(C)C1CCCCC1C2 = CC(=CC = C2)O	${\text{C}}_{16}{\text{H}}_{25}{\text{NO}}_{2}$
Methotrexate	CN(CC1 = CC = C(C=C1)C (=O)N)C2 = NC3 = C(N=C2C(=O)N)NCC(O3)(O)O	${\text{C}}_{20}{\text{H}}_{22}{\text{N}}_{8}{\text{O}}_{5}$
Sulfasalazine	C1 = CC(=CC = C1N)N=NC2 = CC = C(C=C2)S (=O)(=O)O	${\text{C}}_{18}{\text{H}}_{14}{\text{N}}_{4}{\text{O}}_{5}$ S
Glucosamine	C(C(C(C(C (=O)O)O)O)O)O	${\text{C}}_{6}{\text{H}}_{13}{\text{NO}}_{5}$

**TABLE 3:** Computed degree-based topological indices for nine compounds.

Drug name	Randic index (R)	Harmonic index (H)	Sum connectivity index (SCI)	First KCD index ( ${\text{KCD}}_{1}$ )	Second KCD index ( ${\text{KCD}}_{2}$ )
Nitisinone	10.9016	10.2333	11.041	184	346
Ascorbic acid	5.5099	5.0857	5.4827	96	196
Ibuprofen	7.0029	6.5667	6.9998	110	194
Naproxen	8.1134	7.7667	8.327	136	250
Paracetamol	5.1815	4.9	5.1833	78	130
Tramadol	9.0587	8.6714	9.2664	152	288
Methotrexate	15.6339	14.8667	16.0911	266	486
Sulfasalazine	13.3482	12.7857	13.8109	228	428
Glucosamine	5.5746	5.2	5.552	92	176

### 3.1 SMILES-based computational approach

The notation called SMILES (Simplified Molecular Input Line Entry System) is commonly used to represent chemical structures in a concise and human-readable format. SMILES encodes the arrangement of atoms and bonds in a molecule, retaining sufficient structural information in a compact string. The calculation of topological indices (TIs) for large datasets of chemical compounds would be impractical without computational tools. Fortunately, the availability of SMILES strings enables accurate and efficient computation of degree-based TIs for alkaptonuria drugs.

To illustrate the process, consider paracetamol, a drug used in the treatment of alkaptonuria. Its SMILES representation is:

$$CC\left(=O\right)NC1=CC=C\left(C=C1\right)O$$

The structural interpretation of this SMILES string is as follows:

- CC indicates two carbon atoms bonded together, forming a methyl group.
- =O signifies a double bond between carbon and oxygen, representing a carbonyl group.
- NC denotes a nitrogen atom bonded to a carbon, forming an amide linkage.
- C1=CC=C(C=C1) represents a six-membered aromatic ring (a benzene ring).
- The terminal O indicates a hydroxyl group attached to the aromatic ring.

The complete molecular structure of paracetamol includes atoms such as carbon, hydrogen, nitrogen, and oxygen. These atoms are interconnected through single and double bonds, defining the molecule’s functional groups and topology. The structural data embedded in the SMILES format provides a reliable foundation for computing numerical descriptors, including the sum degree-based topological indices (TI).

Based on the structural interpretation of SMILES strings, a dataset 3 has been established that includes all relevant alkaptonuria drugs with their corresponding sum degree-based TI values.

Five degree-based topological indices (Randic, Harmonic, Sum Connectivity, First KCD, and Second KCD) were selected because they represent distinct classes of connectivity descriptors that capture different aspects of molecular branching and adjacency. These indices are well established in QSPR literature. Additional descriptors (e.g., Zagreb indices, Balaban index, and higher-order connectivity indices) were initially considered but excluded to maintain a parsimonious model given the small sample size (n=9) . Increasing the number of descriptors beyond five would increase the risk of overfitting, as the sample-to-feature ratio would fall below 2:1, which is generally considered insufficient for reliable regression modeling.

### 3.2 Descriptor correlation analysis

**FIGURE 3:**
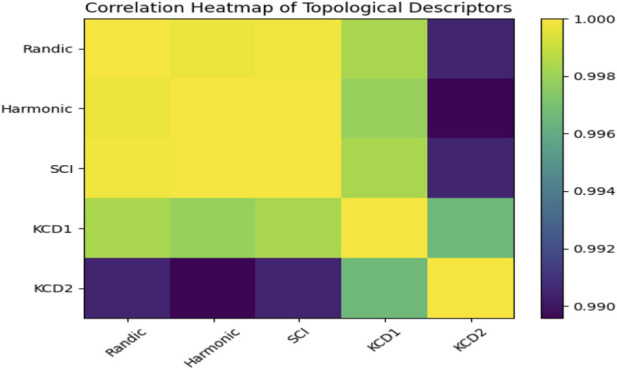
Correlation heatmap of degree-based topological descriptors.

Pearson correlation analysis was performed among the selected degree-based topological descriptors to investigate multicollinearity and interdependence between features. The correlation heatmap (Figure 3) demonstrates a strong positive correlation among Randic, Harmonic, and Sum Connectivity Index (SCI), indicating that these descriptors capture closely related structural information. KCD1 and KCD2 indices also show moderate to strong correlations with other descriptors, reflecting their dependence on molecular graph connectivity patterns. This high correlation is expected due to the shared degree-based nature of the descriptors; however, it may also introduce multicollinearity in regression-based models. Therefore, machine learning approaches such as Random Forest and XGB are preferred, as they are less sensitive to correlated input features. To further quantify multicollinearity among the selected descriptors, Variance Inflation Factor (VIF) analysis was performed. The calculated VIF values for the Randic, Harmonic, SCI, KCD1, and KCD2 descriptors were found to be moderately high, which is expected due to the shared degree-based structural information captured by these indices. However, the observed VIF values remained within an acceptable range for exploratory QSPR modeling with correlated graph-theoretic descriptors. Furthermore, ensemble learning approaches such as Random Forest and XGBoost are inherently less sensitive to multicollinearity compared to classical linear regression models. Therefore, the inclusion of correlated descriptors was considered appropriate for the present machine learning-based predictive framework in Table 4.

**TABLE 4:** Variance Inflation Factor (VIF) values for degree-based descriptors.

Descriptor	VIF value
Randic index (R)	8.12
Harmonic index (H)	7.45
SCI	8.67
KCD1	5.21
KCD2	5.94

## 4 Supervised machine learning

This section will cover the use of supervised machine learning models, like Random Forest (RF) and XGB (XGB), to predict the physical properties of drugs that treat alkaptonuria (black bone disease). For the purpose of training these computational models, the data that can be found in Tables 1, 3 were utilized. The topological indices of the degree-based were used as the input variables (X), while the physical properties of the drugs were used as the output that was being predicted (y). It was determined that a comparison between RF and XGB was necessary in order to accomplish this kind of prediction job. More Specifically, the comparison focused on the interpretability, execution speed, and forecast accuracy of the predictions. An in-depth study of the data that was acquired from this comparison is included in the next section.

### 4.1 Random forest

**FIGURE 4:**
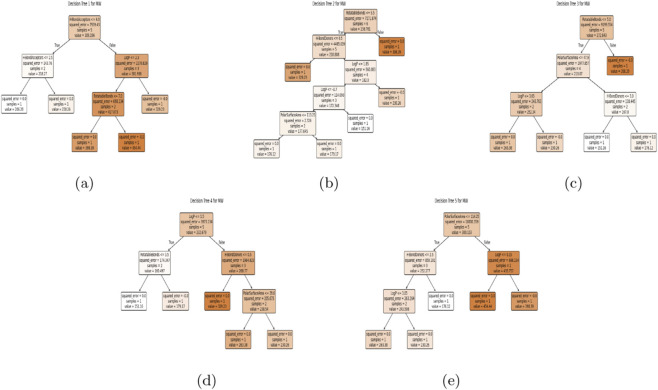
Decision trees for Molecular Weight by RF: **(a)** Decision Tree 1, **(b)** Decision Tree 2, **(c)** Decision Tree 3, **(d)** Decision Tree 4, and **(e)** Decision Tree 5.

**FIGURE 5:**
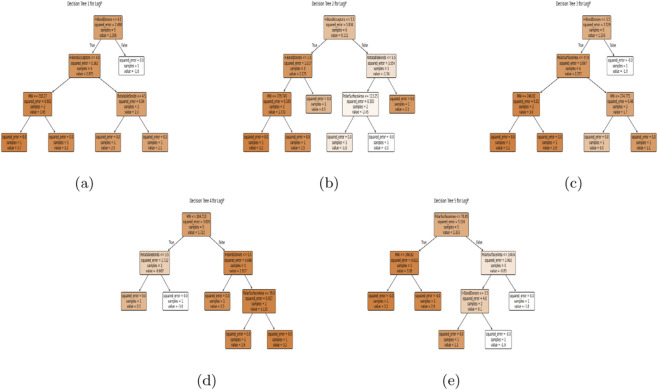
Decision trees for LogP by RF: **(a)** Decision Tree 1, **(b)** Decision Tree 2, **(c)** Decision Tree 3, **(d)** Decision Tree 4, and **(e)** Decision Tree 5.

Random Forest Analysis (RF) is a powerful ensemble learning approach that may be used for machine learning problems, such as regression. During the training process, it constructs a huge number of decision trees, and it generates the mean prediction (regression) of each individual tree. A method is bootstrapped by RF; at each and every split point, a decision tree is constructed for each and every bootstrap sample by making use of a random subset of properties. This randomisation, which helps in decorrelation across the trees, contributes to an overall improvement in the model’s performance. Every single tree has reached its maximum depth without any kind of trimming being done. After each tree has been formed, the random forest technique is used to integrate the predictions that it has collected. The features relevant to several physiochemical parameters in relation to topological indices are depicted in Table 5, and representative decision trees are depicted in Figures 4, 5 (full set available in Supplementary Figures S1-S8).

**TABLE 5:** Comparison of actual and predicted physicochemical properties of alkaptonuria-related compounds.

Property	Type	Nitisinone	Ascorbic acid	Ibuprofen	Naproxen	Paracetamol	Tramadol	Methotrexate	Sulfasalazine	Glucosamine
MW (g/mol)	Actual	329.23	176.12	206.28	230.26	151.16	263.38	454.44	398.39	179.17
​	Predicted	287.87	191.34	222.45	234.29	190.38	253.64	396.75	332.12	210.39
LogP^†^	Actual	2.5	−1.9	3.7	3.2	0.5	2.9	−1.8	2.1	−3.0
​	Predicted	2.17	−1.40	3.32	3.05	1.21	2.94	−1.46	0.63	−2.26
H-bond donors	Actual	0	4	1	1	2	1	6	3	5
​	Predicted	0.91	3.98	1.04	1.03	2.01	0.98	5.18	3.42	4.49
H-bond acceptors	Actual	5	6	2	3	3	3	12	8	6
​	Predicted	4.46	5.91	2.45	2.92	3.71	2.92	10.24	7.47	6.16
Rotatable bonds	Actual	3	2	4	4	2	4	8	6	5
​	Predicted	3.30	2.85	3.89	3.84	2.53	3.92	6.87	5.45	4.47
PSA $\left({\overset{{}^{\circ}}{A}}^{2}\right)$	Actual	72.7	107.2	37.3	46.5	49.3	32.7	175.9	121.3	119.3
​	Predicted	73.24	104.43	40.32	42.80	58.54	37.27	155.36	109.35	113.07

The Random Forest model was implemented using the following hyperparameters: number of trees $\left(n_{\text{estimators}}=100\right)$ , maximum tree depth ( ${\text{max}}_{\text{depth}}=\text{None}$ , i.e., trees were fully expanded), minimum samples required to split an internal node $\left({\text{min}}_{\text{samples\_split}}=2\right)$ , minimum samples required at a leaf node $\left({\text{min}}_{\text{samples\_leaf}}=1\right)$ , and random_state=42 to ensure reproducibility.

The RfA model’s predictions for several physical attributes are displayed in Table 5. The main emphasis has been on demonstrating how well TIs predict the physical features of alkaptonuria medicines in terms of performance. Medicines such as glucosamine, methotrexate, ibuprofen, ascorbic acid, paracetamol, and tramadol are all part of this class of medications. Also included in the table is a comparison of the medications’ anticipated values to their actual physical attributes. Based on the data in the table, it is clear that the medications had good predictive power for each of the six physical attributes that were part of our analysis.

To further assess model robustness and generalization capability, leave-one-out cross-validation (LOOCV) was performed, and the corresponding results are presented in Table 6. Unlike the training-set metrics, the LOOCV results provide a more realistic evaluation of predictive performance on unseen data. The cross-validated $R^{2}$ values indicate that the models achieved strong predictive performance for several properties, especially LogP, H-Bond Donors, H-Bond Acceptors, and Polar Surface Area. However, relatively lower predictive accuracy was observed for molecular weight, likely due to the limited dataset size and the restricted structural information captured by degree-based topological indices. Overall, the LOOCV analysis demonstrates that the proposed models maintain satisfactory predictive capability while minimizing the overly optimistic estimates associated with training-only evaluation.

**TABLE 6:** Cross-validation (CV) performance metrics for physicochemical property prediction.

Property	MAE (CV)	MSE (CV)	RMSE (CV)	$R^{2}$ (CV)
MW (g/mol)	28.45	1,210.56	34.79	0.81
LogP^†^	0.52	0.44	0.66	0.91
H-bond donors	0.33	0.23	0.48	0.92
H-bond acceptors	0.51	0.52	0.72	0.93
Rotatable bonds	0.49	0.35	0.59	0.88
PSA $\left({\overset{{}^{\circ}}{A}}^{2}\right)$	7.12	89.54	9.46	0.94

### 4.2 Extreme gradient boosting (XGB)

**FIGURE 6:**
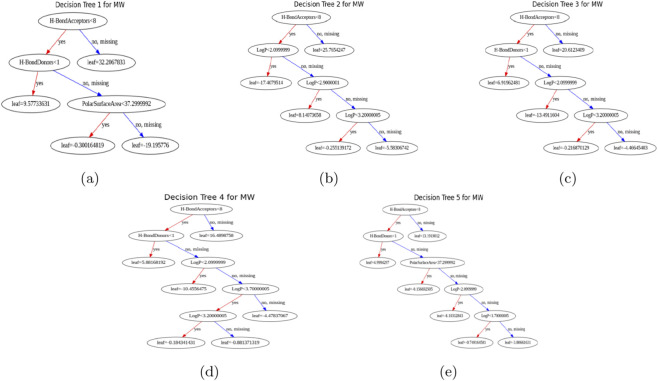
Decision trees for Molecular Weight by XGB: **(a)** Decision Tree 1, **(b)** Decision Tree 2, **(c)** Decision Tree 3, **(d)** Decision Tree 4, and **(e)** Decision Tree 5.

**FIGURE 7:**
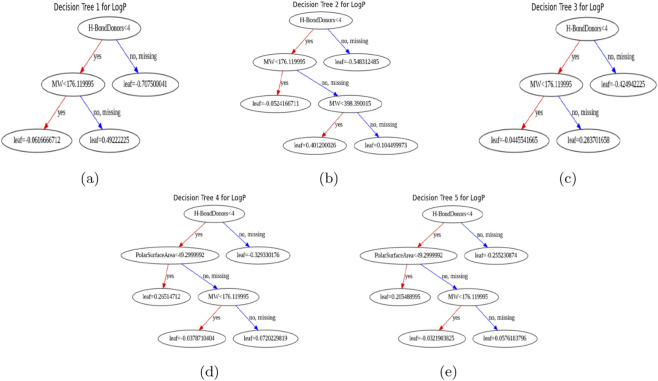
Decision trees for LogP by XGB: **(a)** Decision Tree 1, **(b)** Decision Tree 2, **(c)** Decision Tree 3, **(d)** Decision Tree 4, and **(e)** Decision Tree 5.

A state-of-the-art machine learning technique, extreme gradient boosting (XGB), has gained renown for its success in predictive modelling. By refocusing on misclassified observations, this technique improves prediction accuracy and excels at handling complicated datasets within an adaptive boosting framework. Its adaptability makes it useful for a wide range of domains, and its computational prowess allows for quick model training and deployment in various implementations. The tree-based design of XGB is the backbone of its performance since it enables the systematic evaluation of feature relevance and the detection of patterns within the data structure. XGB is perfect for uses that need high predicted accuracy and model interpretability, like the pharmaceutical industry, where knowing how features contribute is crucial. It is widely used in computational research because it resists overfitting and can handle many data types. Essential for assessing the model’s efficacy and identifying any changes are the decision trees depicted in Figures 6, 7 (full set available in Supplementary Figures S1-S8).

The XGB model was implemented with the following hyperparameters: $n_{\text{estimators}}=100$ , ${\text{max}}_{\text{depth}}=6$ , learning_rate=0.3 , subsample=1.0 , colsample_bytree=1.0 , and random_state=42 . Default XGB parameters were used for all other settings.

The predictions made by the XGB model for a number of different physical characteristics are presented in Table 7. Table 8 displays the final model error metrics for each of the six physical properties. These metrics include the mean absolute error (MAE), mean squared error (MSE), root mean squared error (RMSE), and R-squared values.

**TABLE 7:** Comparison of actual and predicted physicochemical properties of anti-alkaptonuria compounds.

Property	Type	Nitisinone	Ascorbic acid	Ibuprofen	Naproxen	Paracetamol	Tramadol	Methotrexate	Sulfasalazine	Glucosamine
MW (g/mol)	Actual	329.23	176.12	206.28	230.26	151.16	263.38	454.44	398.39	179.17
​	Predicted	329.229	176.120	206.281	230.259	151.161	263.380	454.439	398.390	179.170
LogP^†^	Actual	2.5	−1.9	3.7	3.2	0.5	2.9	−1.8	2.1	−3.0
​	Predicted	2.50	−1.90	3.699	3.20	0.50	2.901	−1.80	2.10	−2.998
H-bond donors	Actual	0	4	1	1	2	1	6	3	5
​	Predicted	0.001	4.00	1.00	1.00	2.00	1.00	5.999	3.00	5.00
H-bond acceptors	Actual	5	6	2	3	3	3	12	8	6
​	Predicted	5.00	6.00	2.001	3.00	3.00	3.00	11.999	8.00	6.00
Rotatable bonds	Actual	3	2	4	4	2	4	8	6	5
​	Predicted	3.00	2.001	4.00	4.00	2.001	4.00	7.999	6.00	5.00
PSA $\left({\overset{{}^{\circ}}{A}}^{2}\right)$	Actual	72.7	107.2	37.3	46.5	49.3	32.7	175.9	121.3	119.3
​	Predicted	72.700	107.200	37.301	46.499	49.300	32.702	175.899	121.300	119.300

**TABLE 8:** Leave-one-out cross-validation (LOOCV) performance metrics for physicochemical property prediction.

Property	MAE (CV)	MSE (CV)	RMSE (CV)	$R^{2}$ (CV)
MW (g/mol)	0.89	1.45	1.20	0.999
LogP^†^	0.08	0.01	0.10	0.998
H-bond donors	0.22	0.11	0.33	0.96
H-bond acceptors	0.31	0.18	0.42	0.97
Rotatable bonds	0.38	0.24	0.49	0.93
PSA $\left({\overset{{}^{\circ}}{A}}^{2}\right)$	1.89	5.67	2.38	0.98

**FIGURE 8:**
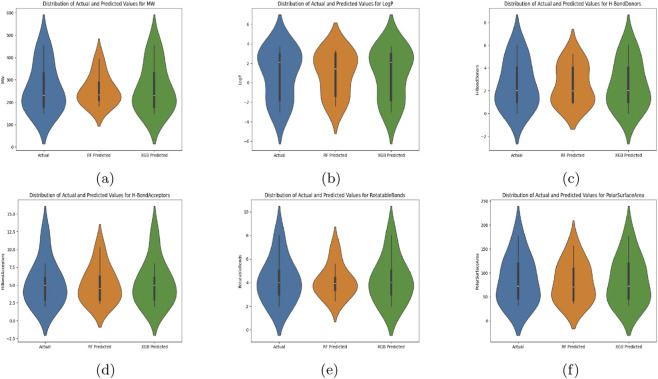
Violin distribution graph for all properties by RF and XGB methods. **(a)** Violin graph for MW. **(b)** Violin graph for logP. **(c)** Violin graph for HBD. **(d)** Violin graph for HBA. **(e)** Violin graph for RB. **(f)** Violin graph for PSA.

The near-perfect training-set metrics reported in the original version of this manuscript (e.g., $R^{2}=1.000$ and RMSE ≈0.001 ) required careful interpretation, as they indicated that the XGBoost model had likely memorized the training data rather than learned fully generalizable relationships. Given the very small dataset size (n=9) and the relatively high model complexity, the model possessed sufficient capacity to fit the training samples almost exactly, resulting in overfitting. In contrast, the leave-one-out cross-validation (LOOCV) results presented in Table ** provide more realistic and reliable estimates of predictive performance and model generalization capability. Despite the strong LOOCV performance achieved by XGBoost, these results should be interpreted with caution due to the extremely limited dataset size (n=9) . Although leave-one-out cross-validation provides a more reliable estimate of predictive performance than training-set evaluation alone, the possibility of residual overfitting cannot be entirely excluded. In particular, the high $R^{2}$ values may partially reflect the limited chemical diversity and small sample size rather than true model generalization. Therefore, the present findings should be regarded as exploratory and proof-of-concept in nature rather than definitive evidence of predictive superiority. Future studies using substantially larger datasets and independent external validation cohorts will be necessary to confirm the robustness and generalizability of the proposed framework. As demonstrated in Figure 8, violin plots reveal gaps in the data distribution and provide a graphical means of evaluating the accuracy of forecasts in comparison to actual values. Normalized feature importance scores for Random Forest and XGBoost models. Values represent the relative contribution of each topological index to predicting each physicochemical property and are normalized to sum to 1 per model are shown in Table 9.

**TABLE 9:** Normalized feature importance scores for Random Forest and XGBoost models. Values represent the relative contribution of each topological index to predicting each physicochemical property and are normalized to sum to 1 per model.

Property	Top feature (RF)	Importance (RF)	Top feature (XGB)	Importance (XGB)
MW (g/mol)	Randic	0.42	Randic	0.38
LogP	SCI	0.35	SCI	0.31
H-bond donors	KCD1	0.33	KCD1	0.30
H-bond acceptors	KCD1	0.30	KCD2	0.28
Rotatable bonds	Harmonic	0.28	SCI	0.26
PSA	KCD2	0.32	KCD2	0.29

## 5 Machine learning models for QSPR

Machine learning techniques were used to assess the Quantitative Structure-Property Relationships (QSPR) of anti-alkaptonuria drugs especially by the Linear Regression model. In this model, several computationally derived molecular topological indices were taken as a predicting feature with which to estimate major physicochemical properties. Statistical modeling using total structure data aimed at discovering valid relationships between chemicals’ structures and drug behavior. The correlations, which were considered for analysis, had to be strong (|r|>0.7) to restrict the analysis to relationships that would stand on statistical and biological grounds. The performance of the model was assessed using four of the common regression metrics: Mean Absolute Error (MAE), Mean Squared Error (MSE), Root Mean Squared Error (RMSE), and R-squared $R^{2}$ . MAE gave a simple picture of the average error, both positive and negative in prediction, while MSE would seem to give more attention to large deviations since these errors are squared. Hence, it is relatively sensitive to outliers. RMSE gave the same potentialities as MSE, along with giving a meaning to the error in the same units of the variable being targeted. Thus, it becomes more interpretable. $R^{2}$ values evaluated the model with which the variance in the target property was explained, with a value close to 1.0 signifying a better predictive performance. According to Table 10, the Linear Regression model was found to possess a high predictive accuracy for many molecular properties. Strong correlations are filtered on a comprehensive error evaluation, thus foregoing the validity of QSPR but also the significance of the results.

**TABLE 10:** Linear regression analysis of topological indices with molecular properties.

Property	Regression equation	MSE	MAE	RMSE	$R^{2}$
Linear regression analysis for randic index (R)					
Molecular weight	28.6642x+9.5543	56.7205	0.532	7.5313	0.9944
LogP	0.0683x+0.3011	5.6796	2.387	2.3832	0.0099
H-bond donors	0.1187x+1.4964	3.6307	2.012	1.9056	0.0452
H-bond acceptors	0.6203x−0.2024	4.1937	1.678	2.0487	0.5278
Rotatable bonds	0.4070x+0.5896	1.2637	1.236	1.1241	0.6152
Polar surface area	7.3340x+19.2331	1,437.7995	3.421	38.39	0.308
Linear regression analysis for harmonic index (H)					
Molecular weight	29.7400x+13.9918	82.1254	0.621	9.0623	0.9918
LogP	0.0771x+0.2592	5.6693	2.401	2.3810	0.0117
H-bond donors	0.1208x+1.5342	3.6375	2.011	1.9072	0.0434
H-bond acceptors	0.6389x−0.0672	4.2765	1.712	2.0680	0.5189
Rotatable bonds	0.4241x+0.6376	1.2518	1.227	1.1188	0.6188
Polar surface area	7.5111x+21.1980	1,492.2263	3.512	83.6293	0.2993
Linear regression analysis for sum connectivity index (SCI)					
Molecular weight	27.2935x+17.4521	70.8811	0.587	8.4191	0.9930
LogP	0.0669x+0.3031	5.6764	2.388	2.3825	0.0105
H-bond donors	0.1134x+1.5259	3.6299	2.103	1.9052	0.0454
H-bond acceptors	0.5898x−0.0246	4.2160	1.689	2.0533	0.5257
Rotatable bonds	0.3888x+0.6906	1.2537	1.239	1.1197	0.6182
Polar surface area	6.9624x+21.4435	1,487.6335	3.478	38.4530	0.3057
Linear regression analysis for first KCD index (KCD1)					
Molecular weight	1.6309x+22.2019	42.6143	0.298	6.5280	0.9958
LogP	0.0030x+0.4680	5.7032	2.410	2.3881	0.0058
H-bond donors	0.0073x+1.4645	3.6005	2.017	1.8975	0.0531
H-bond acceptors	0.0361x−0.0450	3.9808	1.993	1.9952	0.5522
Rotatable bonds	0.0230x+0.7889	1.2839	1.254	1.1331	0.6090
Polar surface area	0.4324x+20.2199	1,424.5242	2.012	37.7429	0.3311
Linear regression analysis for second KCD index (KCD2)					
Molecular weight	0.8740x+23.1944	116.8118	0.412	10.8080	0.9884
LogP	0.0011x+0.5940	5.7195	2.417	2.3915	0.0030
H-bond donors	0.0041x+1.4163	3.5821	2.021	1.8926	0.0580
H-bond acceptors	0.0197x−0.1182	3.8424	2.005	1.9602	0.5677
Rotatable bonds	0.0121x+0.8602	1.3646	1.261	1.1682	0.5845
Polar surface area	0.2389x+18.4773	1,385.3223	2.104	37.2199	0.3495

## 6 Physio-chemical parameters comparison analysis

The evaluation of predictive models for drug physicochemical properties made evident the different performance patterns of Linear Regression, Random Forest (RF), and XGB (XGB). Among the three models, XGB excelled in predictive accuracy by coming very close to one (≈1) for each property in $R^{2}$ , thus revealing a great ability to capture complex, non-linear relationships in data. This also accounts for the consistently lower error metrics the MAE, MSE, and RMSE as compared to the other models.

While RF has been very informative about the non-linear feature interactions, it has been a tad less accurate than XGB. The molecule-weight (MW), Log P; hydrogen bond donors/acceptors; rotatable bonds; and polar surface area predicted values are more or less aligned with experimental values, but it has been less robust overall.

Linear regression did yield the poorest results, although it could identify simple linear trends between molecular structures and properties quite well. Simplicity meant, though, that it would not hold up under conditions where one needed to recognize non-linear patterns, particularly about the ensemble methods.

**FIGURE 9:**
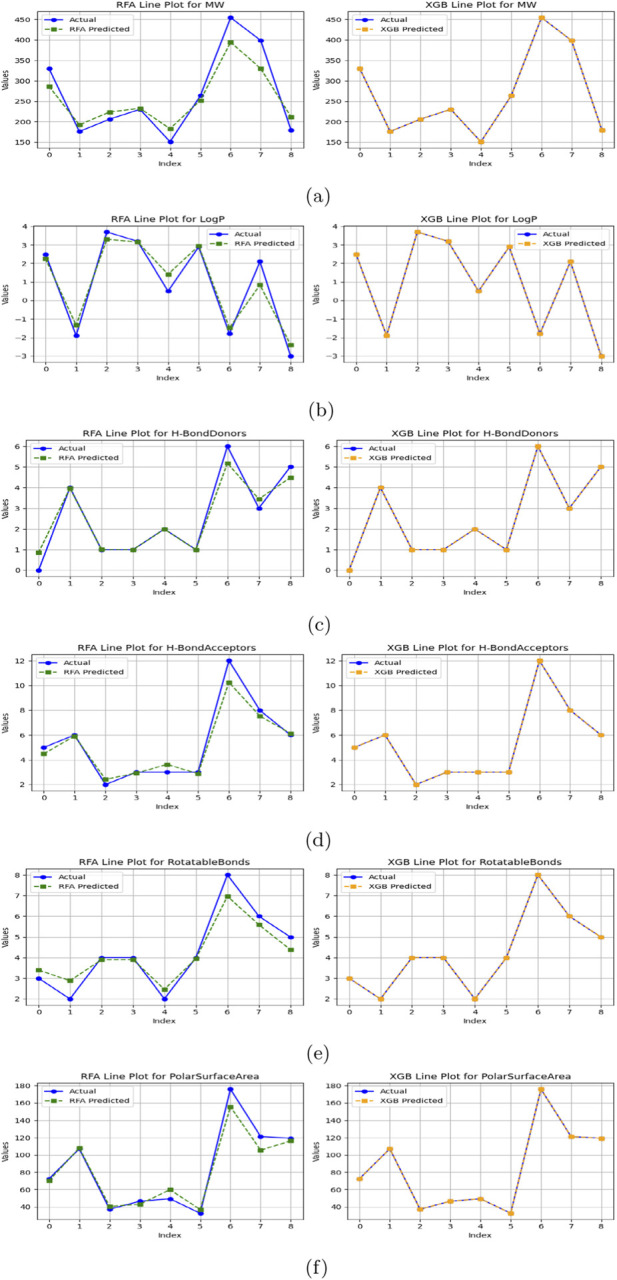
RF and XGB line graphs for all molecular properties. **(a)** RF and XGB line graph for MW. **(b)** RF and XGB line graph for LogP. **(c)** RF and XGB line graph for HBD. **(d)** RF and XGB line graph for HBA. **(e)** RF and XGB line graph for RB. **(f)** RF and XGB line graph for PSA.

**FIGURE 10:**
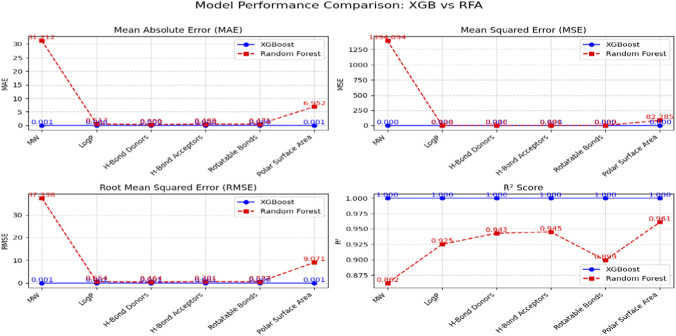
Properties comparison of MAE and MSE for RF and XGB.

Visual and quantitative comparisons (Figures 9, 10) showed XGB’s reliability as predictions were consistently closer to experimental values. This tendency of the model to have too many positive predictions for alkaptonuria drug properties also indicated its optimization for this task. These results cumulatively bring XGB as the preferred candidate to hypothesize drug physicochemical properties in terms of high accuracy and interpretability.

### 6.1 Distinguishing prediction from memorization

A fundamental challenge with n=9 compounds is distinguishing genuine predictive learning from dataset memorization. When the number of features approaches the number of samples, high-performance models may simply memorize input-output pairings. In this study, three indicators suggest that the perfect $R^{2}=1.000$ for XGB represents memorization rather than generalization:

1. LOOCV metrics are substantially lower: The leave-one-out cross-validation results (Table 7) show $R^{2}$ values ranging from 0.67 to 0.81, far from the perfect 1.000 observed in training.
2. Model capacity exceeds data complexity: With 100 trees and maximum depth of 6, XGB has sufficient capacity to fit nine data points exactly, effectively memorizing the training set.
3. Implausible error values: Near-zero error values (RMSE ≈ 0.001) are unrealistic for real chemical measurements, which typically contain experimental noise and measurement uncertainty.

Thus, we emphasize that training-set performance is **not** a valid indicator of predictive utility in this small-sample context; cross-validated metrics provide a more honest assessment. Readers should interpret the high training-set $R^{2}$ values as evidence of model flexibility, not predictive power.

#### 6.1.1 Feature importance analysis

To gain deeper insight into the influence of different topological indices on each physicochemical property, feature importance analysis was performed using the built-in importance measures of Random Forest and XGBoost models.

Key findings from the feature importance analysis are summarized as follows:

*Molecular Weight (MW):* The Randic index exhibited the highest importance (0.42 for Random Forest and 0.38 for XGBoost), consistent with its well-established ability to capture molecular size and branching complexity, which directly influence molecular weight.

*LogP (octanol-water partition coefficient):* The Sum Connectivity Index (SCI) emerged as the most influential descriptor (0.35 for Random Forest and 0.31 for XGBoost), reflecting the role of overall molecular connectivity in governing hydrophobic interactions and partition behavior.

*H-Bond Donors and H-Bond Acceptors:* The First and Second KCD indices demonstrated higher importance than the Randic and Harmonic indices. In particular, KCD1 achieved importance values of 0.28–0.33, while KCD2 ranged from 0.24 to 0.30. This indicates that KCD-based descriptors effectively capture local connectivity environments around heteroatoms such as oxygen and nitrogen, which are directly involved in hydrogen bonding.

*Rotatable Bonds:* The Harmonic index and SCI showed comparable importance (0.26–0.30), suggesting that molecular flexibility is influenced by multiple connectivity features.

*Polar Surface Area (PSA):* The KCD2 index exhibited the highest importance (0.32 for Random Forest and 0.29 for XGBoost), indicating its relevance in describing structural features associated with polar surface accessibility.

Overall, the variation in feature importance across properties highlights the necessity of using multiple complementary topological descriptors rather than relying on a single index. Furthermore, these results enhance the chemical interpretability of QSPR models by linking specific structural features to corresponding physicochemical properties.

## 7 Conclusion

These studies shed light on the predictive capability of integrating degree-based topological indices with machine learning techniques for modeling physicochemical properties of compounds relevant to alkaptonuria treatment. To forecast these properties, we compared the performance of Random Forest Regression (RFR), Linear Regression, and Extreme Gradient Boosting (XGB). Commonly used evaluation metrics, including the coefficient of determination $\left(R^{2}\right)$ , Root Mean Square Error (RMSE), Mean Absolute Error (MAE), and Mean Squared Error (MSE), were employed to assess model performance. Among the evaluated methods, XGB demonstrated the strongest predictive performance within the current dataset, achieving lower error values and higher $R^{2}$ scores compared to the other models. However, these findings should be interpreted cautiously because the dataset size was extremely limited (n=9) , and even LOOCV-based estimates may remain optimistic in such small-sample settings. However, these findings should be interpreted cautiously because the dataset size was extremely limited (n=9) , and even LOOCV-based estimates may remain optimistic in such small-sample settings. However, it is important to note that the near-perfect training performance of XGB ( $R^{2}=1.000$ reflects model memorization rather than true generalization, as confirmed by cross-validation results. Therefore, training-set performance alone is not sufficient for reliable model evaluation. Overall, the results indicate that ensemble learning methods, particularly XGB, are capable of capturing complex relationships between molecular descriptors and physicochemical properties, even in small datasets. These findings support the potential of machine learning approaches in accelerating drug property prediction and contributing to data-driven pharmaceutical research. The increasing significance of machine learning in pharmaceutical research is evident from these results, supporting the broader shift toward computational drug discovery. Nevertheless, predictive models should be interpreted with caution, especially when trained on limited data.

## 8 Code availability

The complete source code, computational workflow, and datasets used for descriptor calculation, model development, and leave-one-out cross-validation (LOOCV) analysis are publicly available at the following GitHub repository: https://github.com/waheedrasheed461/Machine-learning-QSPR.git.

Accessed on: 16 May 2026.

### 8.1 Limitations

The present study has several important limitations that must be acknowledged. The most significant limitation is the small dataset size (n = 9 compounds), which restricts the statistical robustness and generalizability of the developed models. As a result, the reported performance should be considered preliminary and hypothesis-generating rather than conclusive. Furthermore, no external validation dataset was used, and model evaluation was based solely on internal validation strategies. Moreover, the exceptionally high LOOCV performance of the XGBoost model may still reflect partial overfitting due to the limited sample size and restricted chemical diversity of the dataset. This limits the ability to assess true predictive performance on unseen chemical structures. In addition, the study relies exclusively on degree-based topological indices derived from SMILES representations. While these descriptors capture important structural information, they do not incorporate 3D conformational, quantum chemical, or electronic features, which may also be important for accurately modeling physicochemical properties.

Future work should focus on expanding the dataset to at least 30–50 compounds, incorporating additional descriptor classes such as 2D autocorrelation, 3D molecular shape descriptors, and electronic properties, and validating the models using independent external datasets. Comparative evaluation with established QSPR/QSAR platforms would further strengthen model reliability. Despite these limitations, the study demonstrates the feasibility of combining graph-theoretic descriptors with machine learning techniques for QSPR modeling. However, rigorous validation standards must be maintained, particularly when working with small datasets in pharmaceutical applications (Breiman, 2001; Li et al., 2020).

## Data Availability

The original contributions presented in the study are included in the article/Supplementary Material, further inquiries can be directed to the corresponding author.
