# Supplementary material for: SMILES-based degree molecular descriptors and machine learning for QSPR modeling of anti-alkaptonuria drugs
Source: Front Chem. 2026 Jun 15;14:1845927. doi: 10.3389/fchem.2026.1845927 (PMC13310881; doi:10.3389/fchem.2026.1845927)
Supplement: Supplementary file 1 [file DataSheet1.pdf]

This supplementary information contains the complete set of decision tree figures for Random Forest (Figures S1-S4) and XGBoost (Figures S5-S8) across all six physicochemical properties. For clarity and brevity, only representative trees for Molecular Weight and LogP are presented in the main manuscript.

Random Forest Decision Trees

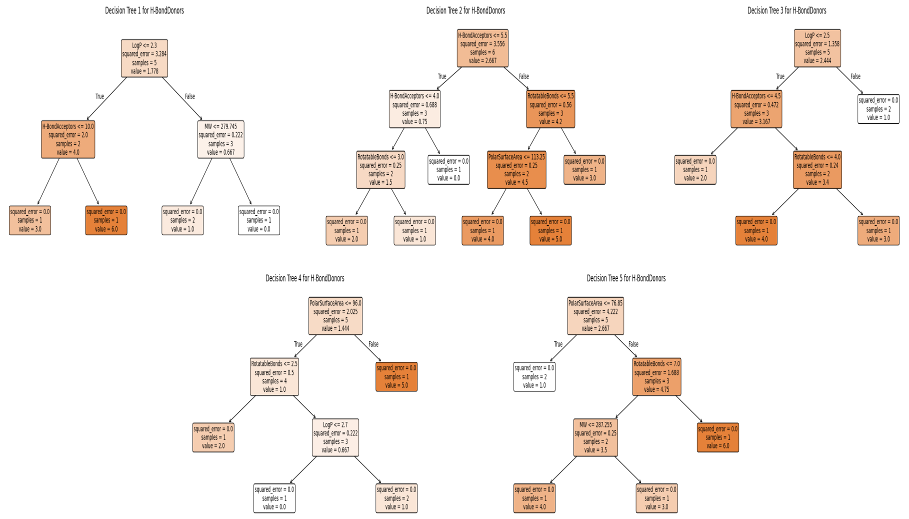

Figure 1: S1: Decision trees for H-Bond Donors using Random Forest

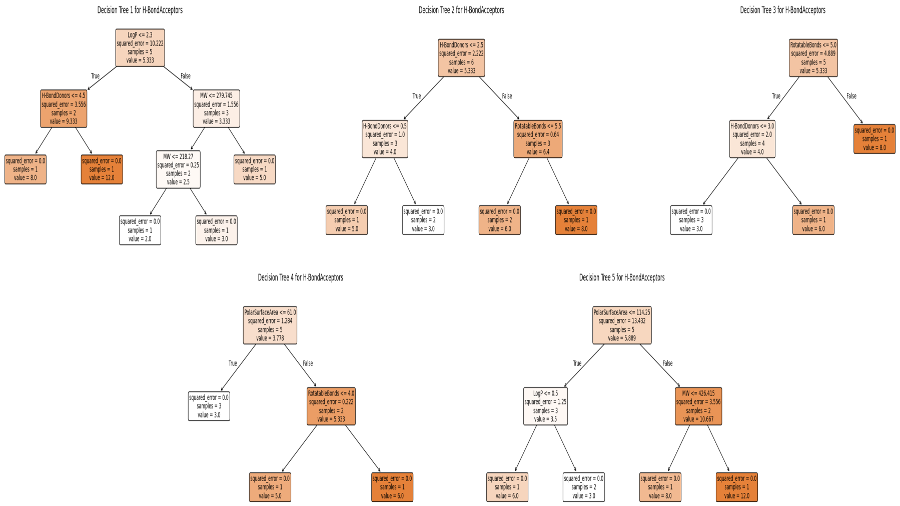

Figure 2: S2: Decision trees for H-Bond Acceptors using Random Forest

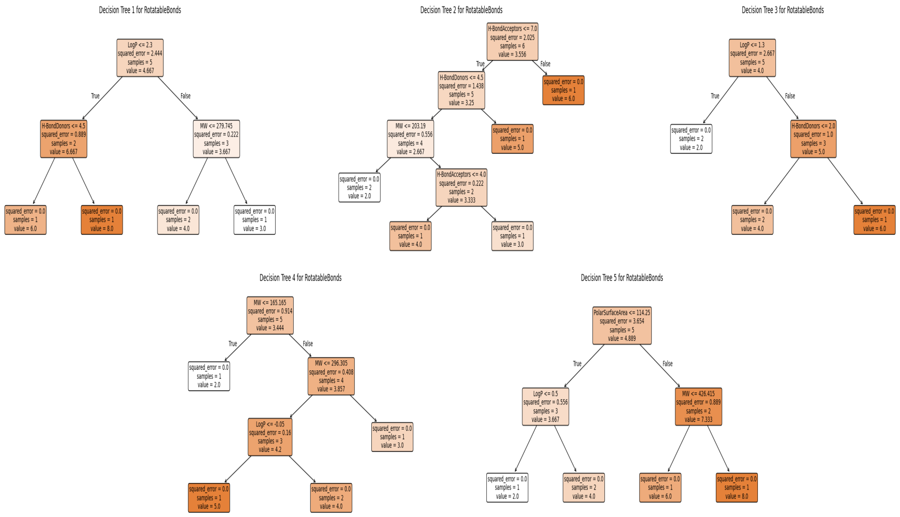

Figure 3: S3: Decision trees for Rotatable Bonds using Random Forest

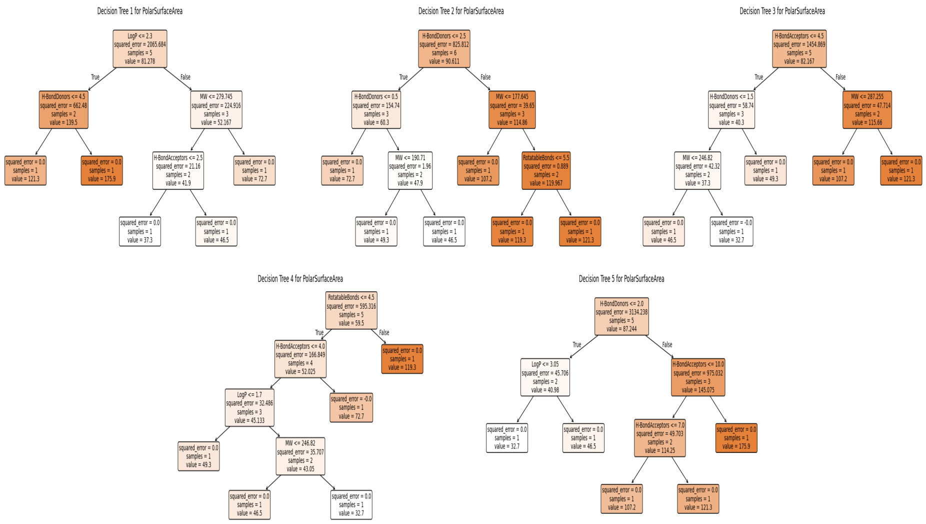

Figure 4: S4: Decision trees for Polar Surface Area using Random Forest

XGBoost Decision Trees

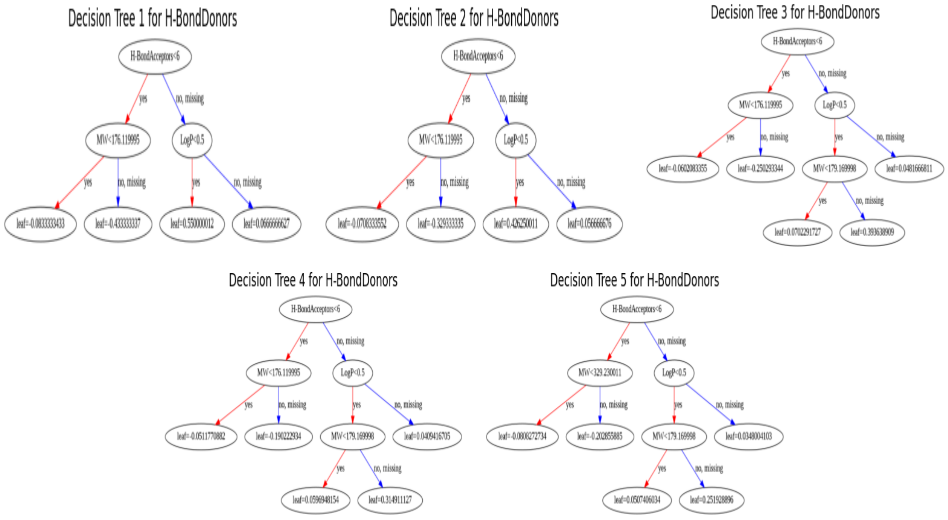

Figure 5: S5: Decision trees for H-Bond Donors using XGBoost

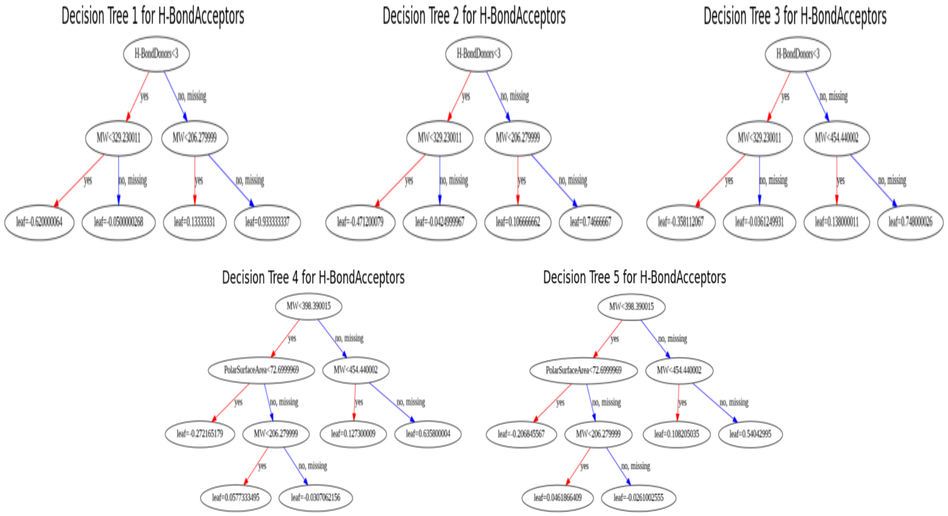

Figure 6: S6: Decision trees for H-Bond Acceptors using XGBoost

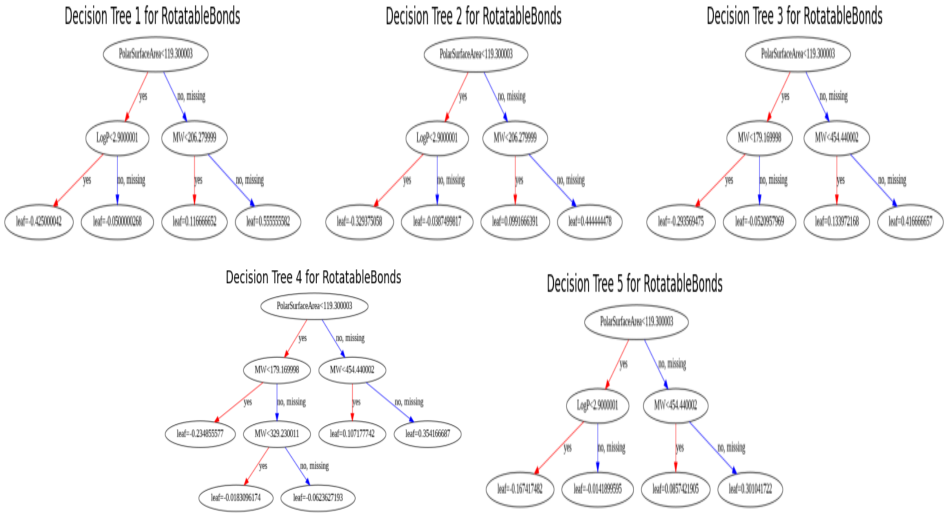

Figure 7: S7: Decision trees for Rotatable Bonds using XGBoost

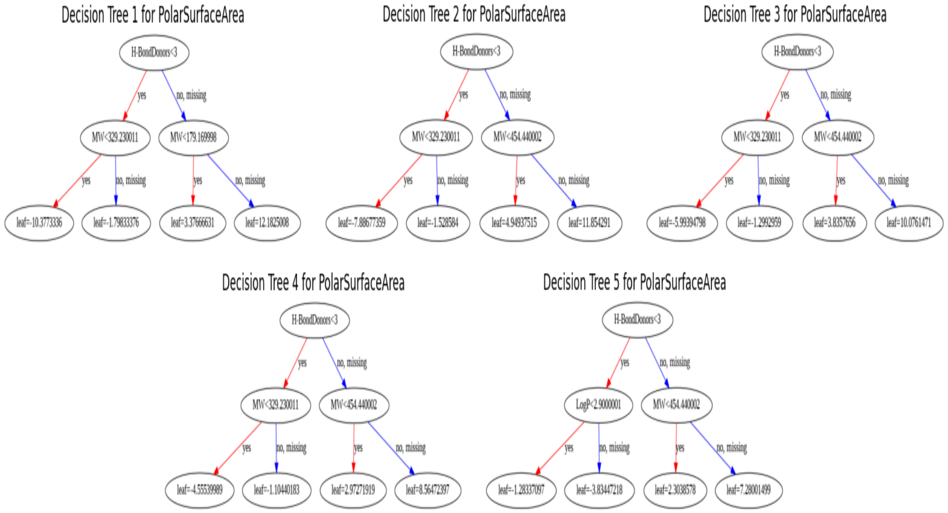

Figure 8: S8: Decision trees for Polar Surface Area using XGBoost
